# Glial neurovascular unit protein dysregulation and risk of idiopathic intracranial hypertension: A systematic review and meta-analysis

**DOI:** 10.3205/000358

**Published:** 2026-06-03

**Authors:** Bhoomika Arora, Manabesh Nath, Poorvi Tangri, Pradeep Kumar, Rajesh Kumar Singh, Gyaninder Pal Singh, Deepti Vibha, Achal Kumar Srivastava, Divyani Garg, Ayush Agarwal, Divya M. R., Vishnu V. Y., Awadh Kishor Pandit

**Affiliations:** 1 Department of Neurology, All India Institute of Medical Sciences, New Delhi, India; 2 Clinical Research Unit, All India Institute of Medical Sciences, New Delhi, India; 3 Department of Neuroanaesthesiology, All India Institute of Medical Sciences, New Delhi, India

**Keywords:** idiopathic intracranial hypertension, glial neurovascular unit, aquaporin-4, fibrinogen, neurofilament-light chain, GFAP, biomarkers, meta-analysis

## Abstract

**Background:**

Idiopathic intracranial hypertension is a neurological disorder of unclear etiology that primarily affects young adults. Although several therapeutic options are available, many patients continue to experience persistent visual impairment and headaches, suggesting that key disease mechanisms remain poorly understood. Emerging evidence implicates dysfunction of the glial neurovascular unit (gNVU) in the pathogenesis of IIH.

**Objective:**

To systematically evaluate the association between alterations of gNVU-related proteins, specifically aquaporin-4, glial fibrillary acidic protein, fibrinogen, and neurofilament light chain, with the risk of IIH.

**Methods:**

A comprehensive literature search of PubMed, EMBASE, Cochrane Library, Scopus, and Web of Science was conducted through August 2025. Studies comparing patients with IIH to control participants and reporting gNVU-related biomarkers in cerebrospinal fluid, plasma, serum, or brain tissue were included. Data extraction and quality assessment were performed independently using the Newcastle–Ottawa Scale. Pooled standardized mean differences with 95% confidence intervals were calculated using random-effects models.

**Results:**

Ten studies comprising 327 patients with IIH and 216 controls met the inclusion criteria, with seven studies contributing to quantitative synthesis. Levels of CSF Nf-L and plasma fibrinogen were significantly higher in IIH patients, indicating neuroaxonal injury and a hypercoagulable state (Nf-L: SMD=0.78, 95% CI 0.51–1.05; fibrinogen: SMD=0.66, 95% CI 0.08–1.23). Findings for AQP4 and GFAP were inconsistent across studies. Higher BMI was also associated with an increased risk of IIH (SMD=0.80, 95% CI 0.17–1.44). Study quality did not significantly influence effect estimates.

## Zusammenfassung

**Hintergrund:**

Die idiopathische intrakranielle Hypertension ist eine neurologische Erkrankung unklarer Ätiologie, die vor allem junge Erwachsene betrifft. Obwohl mehrere therapeutische Optionen verfügbar sind, erleben viele Patienten weiterhin anhaltende Sehstörungen und Kopfschmerzen, was darauf hindeutet, dass die wesentlichen Krankheitsmechanismen noch schlecht verstanden sind. Neue Erkenntnisse deuten auf eine Funktionsstörung der glialen neurovaskulären Einheit (gNVU) bei der Pathogenese der IIH hin.

**Ziel:**

Systematische Bewertung des Zusammenhangs zwischen Veränderungen von gNVU-bezogenen Proteinen, insbesondere Aquaporin-4, glialem fibrillärem saurem Protein, Fibrinogen und Neurofilament-Leichtkette, und dem Risiko für IIH.

**Methoden:**

Es wurde eine umfassende Literatursuche in PubMed, EMBASE, Cochrane Library, Scopus und Web of Science bis August 2025 durchgeführt. Eingeschlossen wurden Studien, die Patienten mit IIH mit Kontrollteilnehmern verglichen und gNVU-bezogene Biomarker in Liquor, Plasma, Serum oder Gehirngewebe berichteten. Die Datenerhebung und Qualitätsbewertung wurden unabhängig unter Verwendung der Newcastle–Ottawa-Skala durchgeführt. Zusammengefasste standardisierte Mittelwertdifferenzen mit 95%-Konfidenzintervallen wurden unter Verwendung von Random-Effects-Modellen berechnet.

**Ergebnisse:**

Zehn Studien mit insgesamt 327 Patienten mit IIH und 216 Kontrollen erfüllten die Einschlusskriterien, wobei sieben Studien zur quantitativen Synthese beitrugen. Die Werte von CSF Nf-L und Plasmafibrinogen waren bei IIH-Patienten signifikant höher, was auf eine neuroaxonale Schädigung und einen hyperkoagulierbaren Zustand hinweist (Nf-L: SMD=0,78, 95% CI 0,51–1,05; Fibrinogen: SMD=0,66, 95% CI 0,08–1,23). Die Befunde für AQP4 und GFAP waren zwischen den Studien inkonsistent. Ein höherer BMI war ebenfalls mit einem erhöhten Risiko für IIH verbunden (SMD=0,80, 95% CI 0,17–1,44). Die Studienqualität hatte keinen signifikanten Einfluss auf die Effektgrößen.

**Schlussfolgerungen:**

Erhöhte Spiegel von Nf-L und Fibrinogen sind mit einem erhöhten Risiko für IIH assoziiert und stützen die Beteiligung von neuroaxonaler Schädigung und Hyperkoagulabilität an der Pathophysiologie der Erkrankung. Größere, multizentrische Studien unter Verwendung standardisierter Biomarker-Assays, Bildgebung und klinischem Phänotyping sind erforderlich, um diese Ergebnisse zu validieren und die Entwicklung verbesserter diagnostischer und therapeutischer Strategien zu unterstützen.

## Graphical abstract


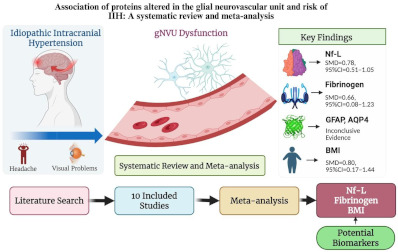


**Graphical abstract created in BioRender by Tangri **[1]**.**

## Introduction

According to the International Classification of Headache Disorders, 3^rd^ edition (ICHD-3, 2018) issued by the International Headache Society, idiopathic intracranial hypertension (IIH) is classified under headaches attributed to nonvascular intracranial disorders, specifically headaches related to increased cerebrospinal fluid (CSF) pressure. Clinically, most patients with IIH present with headache (approximately 84%), transient visual disturbances (68%), pulsatile tinnitus, and dizziness (52%). Additional symptoms such as neck pain, back pain, and cognitive difficulties are also reported in a subset of patients [2].

IIH is considered a rare disorder, with an estimated incidence ranging from 0.2 to 2 per 100,000 individuals in the general population aged 25–36 years. It occurs more frequently in women than men (7.7 vs. 1.6 per 100,000). However, the rising prevalence of obesity and sedentary lifestyles has been accompanied by a marked increase in IIH incidence. Between 2002 and 2016, the incidence of IIH among women increased by 118% [3], [4]. As a result, IIH has a substantial impact on quality of life, visual function, and work capacity, particularly in young adults.

Identifying the underlying cause of IIH remains critical. Although acetazolamide is the most commonly prescribed medication, a recent Cochrane review concluded that there is insufficient evidence to either support or refute its effectiveness [5]. Moreover, treatment with acetazolamide or topiramate often leaves patients with persistent symptoms and abnormal pulsatile intracranial pressure. While CSF diversion procedures can improve visual outcomes, they are frequently associated with ongoing headaches and high rates of shunt revision [6], [7], [8]. Overall, current treatment strategies focus on lowering intracranial pressure rather than addressing the root cause of the disease.

IIH remains a disorder with an unclear etiology, encompassing multiple hypotheses and treatment approaches, including medical, surgical, and lifestyle interventions. Recent work by Eide and colleagues has highlighted the potential role of dysfunction within the glial neurovascular unit (gNVU) in IIH pathophysiology [9]. Building on these observations, the present study evaluates gNVU-related proteins and their association with IIH risk.

The gNVU is a complex and highly specialized structure composed of endothelial cells forming tight junctions, pericytes that regulate capillary stability, and supporting cells including astrocytes, neurons, and microglia. Together, these components maintain blood–brain barrier integrity and neurovascular coupling. In IIH, elevated intracranial pressure is thought to arise from pathological cellular and molecular alterations within this unit. Eide et al. demonstrated pronounced structural abnormalities in IIH, including increased glial fibrillary acidic protein (GFAP) expression, patchy astrogliosis, disruption of astrocytic architecture, basement membrane degeneration, loss of pericyte processes, and blood–brain barrier breakdown with leakage of blood-derived proteins such as fibrinogen [8]. Earlier work also linked astrogliosis in IIH with increased aquaporin-4 (AQP4) expression, suggesting impaired water regulation in the central nervous system [7].

Neuronal injury within the gNVU can be assessed using neurofilament light chain (NfL), a cytoskeletal protein essential for axonal integrity. Elevated CSF and serum NfL levels have been shown to correlate with the severity of papilledema and may predict poor visual outcomes in patients with suspected IIH [10].

Against this background, this systematic review and meta-analysis were undertaken to evaluate the association between altered expression of gNVU-related proteins and the risk of idiopathic intracranial hypertension.

## Materials and methods

### Literature search

This systematic review and meta-analysis were conducted in accordance with the Preferred Reporting Items for Systematic Reviews and Meta-Analyses (PRISMA) guidelines [11]. The study protocol was prospectively registered in the PROSPERO database (Registration ID: CRD420251039144). A comprehensive literature search was performed across PubMed, EMBASE, the Cochrane Library, Scopus, and Web of Science, covering all relevant studies published up to 30 August 2025.

The search strategy combined Medical Subject Headings (MeSH) and free-text terms related to idiopathic intracranial hypertension, including “idiopathic intracranial hypertension,” “IIH,” and “pseudotumor cerebri,” with terms related to glial neurovascular unit–associated proteins, such as “fibrinogen,” “fibrin,” “neurofilament,” “neurofilament light,” “complement proteins,” “aquaporin-4 (AQP4),” “CD68,” and “glial fibrillary acidic protein (GFAP)”.

Only studies conducted in human subjects and published in the English language were included, with no restrictions on publication year. In addition, the reference lists of all included articles, relevant reviews, and prior meta-analyses were manually screened to identify any additional eligible studies.

### PICO of the study

**Population:** Individuals who were clinically diagnosed with IIH and had their CSF or plasma biomarkers (aquaporin, GFAP, fibrinogen, or neurofilament-L) levels assessed were included. 

**Intervention/exposure:** Observation of altered protein levels of the glial neurovascular unit (aquaporin, GFAP, fibrinogen, neurofilament-L)

**Comparator(s)/control(s):** Individuals who were not IIH patients were included in this group [2], [3], [4], [5], [9].

**Primary outcome(s):** Association between various proteins of the glial neurovascular unit (aquaporin, GFAP, neurofilament-L, or fibrinogen) with the risk of developing idiopathic intracranial hypertension.

**Secondary outcome(s):** Association of body mass index with the risk of developing idiopathic intracranial hypertension.

**Study selection:** Eligible studies were selected in the systematic review and meta-analysis if they met all of the following inclusion criteria:

1. Studies comparing IIH cases with non-IIH controls will be included.
2. Studies including humans as subjects
3. Studies reporting levels of proteins associated with the astroglial neurovascular unit in IIH, in terms of mean ± standard deviation or median, range, and/or interquartile range, for comparison between cases and controls

Studies were excluded if they met any of the following criteria:

1. Studies such as reviews, conference abstracts, and preprints
2. Studies including animals as subjects
3. Studies with insufficient data
4. Studies in languages other than English or that are not translatable
5. Duplicate publications
6. Studies measuring biomarker levels in a single cohort of participants with no comparator

### Data extraction

Two investigators (BA and MN) independently screened studies retrieved from the electronic databases by reviewing titles and abstracts based on predefined eligibility criteria. Duplicate records were removed, and study selection was further refined using Rayyan software in accordance with the inclusion and exclusion criteria [12].

For each eligible study, relevant data were extracted using a standardized data collection form. Extracted information included the first author’s name, year of publication, country, study population, mean age, sex distribution, sample size (number of IIH cases and controls), and mean values with standard deviations of protein levels in both patients and controls, including GFAP, AQP4, neurofilament light chain, and fibrinogen. Outcome measures were summarized as means and standard deviations for quantitative analysis.

Any disagreements between reviewers were resolved through discussion and consensus among all authors. When required data were missing or unclear, attempts were made to contact the corresponding authors by email on two separate occasions to obtain the necessary information.

### Quality assessment

The methodological quality and risk of bias of all studies included in the systematic review and meta-analysis were independently assessed by two authors (BA and MN) using the Newcastle–Ottawa Scale (NOS). The NOS evaluates study quality across three domains: selection, comparability, and exposure. Scores range from 0 to 9, with studies classified as low quality (0–3), moderate quality (4–6), or high quality (7–9). Any discrepancies in quality assessment were resolved through discussion, with final adjudication by the corresponding author (AKP).

### Publication bias assessment

Publication bias was assessed using Begg’s funnel plot analysis, and funnel plot asymmetry was formally evaluated with Egger’s linear regression test where sufficient data were available.

### Statistical analysis

A meta-analysis was performed only for proteins with reported mean and standard deviation, or median and interquartile range, in at least one study. Data reported as median and range were converted to mean and standard deviation. Forest plots and analyses were generated using STATA 13.1. Heterogeneity was assessed using I² values, with <50% indicating low and >50% indicating moderate-to-high heterogeneity. Depending on heterogeneity, either a fixed-effect model (Mantel–Haenszel, I²<50%) or a random-effects model (DerSimonian–Laird, I²>50%) was applied [9]. Between-study heterogeneity was further evaluated via meta-regression of study quality scores and effect sizes. Sensitivity analyses, removing one study at a time, assessed the influence of individual studies and potential selection bias. Statistical significance was set at p<0.05.

## Results

### Literature search

**Figure 1:**
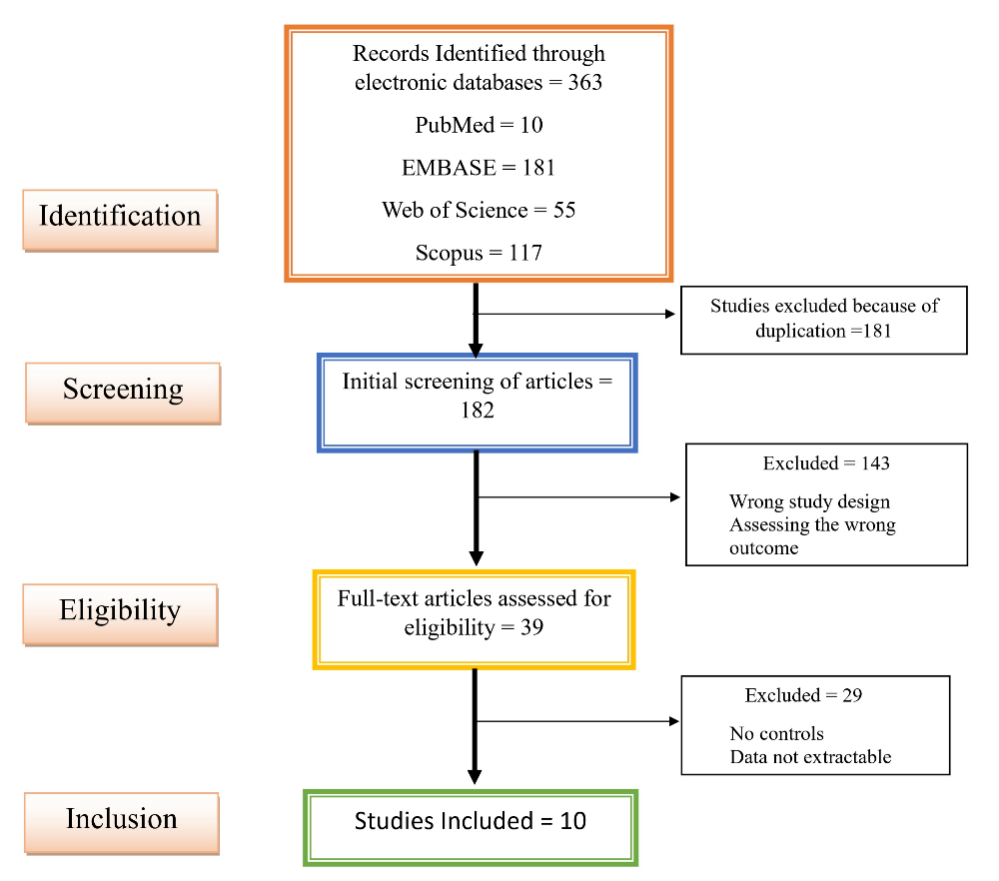
PRISMA Flow Diagram for study search, selection, and inclusion process Adapted from Page et al. [11], licensed under CC BY 4.0 (https://creativecommons.org/licenses/by/4.0/)

Figure 1 (Fig. 1) shows the PRISMA flowchart for the included studies [7]. The initial search across electronic databases identified 363 articles (117 from Scopus, 181 from EMBASE, 55 from Web of Science, and 10 from PubMed). After removing 181 duplicates, 182 articles were screened against the inclusion and exclusion criteria, resulting in the exclusion of 172 studies. The remaining 10 articles were included in the analysis. Excluded studies primarily lacked a comparator or had insufficient data for analysis.

### Characteristics of eligible studies

The included studies in this systematic review were published between 1997 and 2022 and were all in English. Sample sizes ranged from 20 to 128 participants. Diagnostic criteria for IIH varied across studies, reflecting changes from 1985 to 2013. Table 1 (Tab. 1) summarizes the characteristics of studies included in the meta-analysis examining the relationship between glial neurovascular proteins and IIH risk.

Four studies (Hasan-Olive et al., Doppler et al., Eide et al., and Eide et al.) reported AQP4 levels in IIH patients and controls [8], [9], [13], [14]. Comparators differed across studies, including brain tissue from epilepsy resections, elective cerebral aneurysm clipping, parenchymal tumor resections, and lumbar puncture for unrelated diagnostic purposes (Doppler et al.). Quantitative meta-analysis of AQP4 was not possible because Hasan-Olive et al. included patients from Eide et al. and Doppler et al. measured AQP4 in CSF via ELISA, whereas other studies assessed tissue levels using histopathology, making results incomparable.

Two studies (Uzun et al. and Dhunguna et al.) found no AQP4 antibodies in the CSF of IIH patients, suggesting anti-AQP4 antibodies are unlikely to play a causal role. However, small sample sizes mean a role in a subset of patients cannot be excluded [15], [16].

GFAP, a CNS astrocyte protein, was reported in two studies (Eide et al. and Engel et al.). Engel et al. used highly sensitive SiMoA technology to measure CSF and serum GFAP, while Eide et al. assessed GFAP immunoreactivity in brain biopsy samples. Differences in methodology prevented direct comparison [9], [17].

Plasma fibrinogen was reported as elevated in IIH patients in three studies (Kessler et al., Sussman et al., and Hannerz et al.) [18], [19], [20]. Neurofilament light chain (NF-L) was investigated in CSF by three studies (Engel et al., Svart et al., and Knoche et al.), with levels measured using single-molecule array technology [10], [17], [21].

### Primary outcome: Association of glial neurovascular unit proteins with idiopathic intracranial hypertension

We conducted a meta-analysis examining the association between glial neurovascular unit proteins AQP4, GFAP, NF-L, and fibrinogen and the risk of developing IIH. Ten studies were initially identified, comprising 327 IIH cases and 216 controls. Due to methodological differences and non-comparable data, seven studies were included in the quantitative analysis. AQP4 and GFAP data could not be pooled, as these studies employed different specimen types, measurement techniques, and reporting methods.

**Figure 2:**
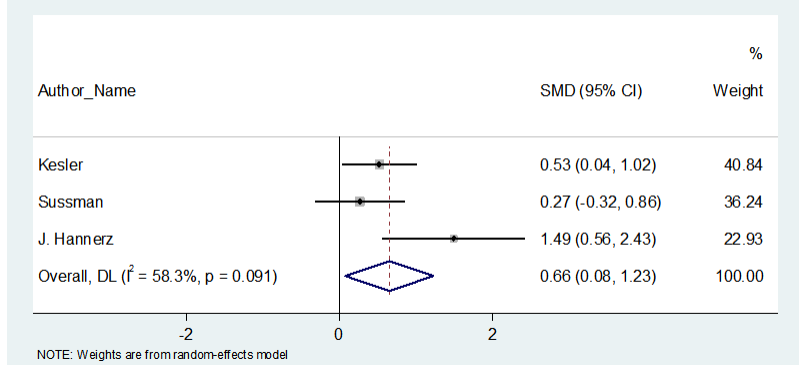
Forest plot showing the association between fibrinogen and the risk of IIH

**Figure 3:**
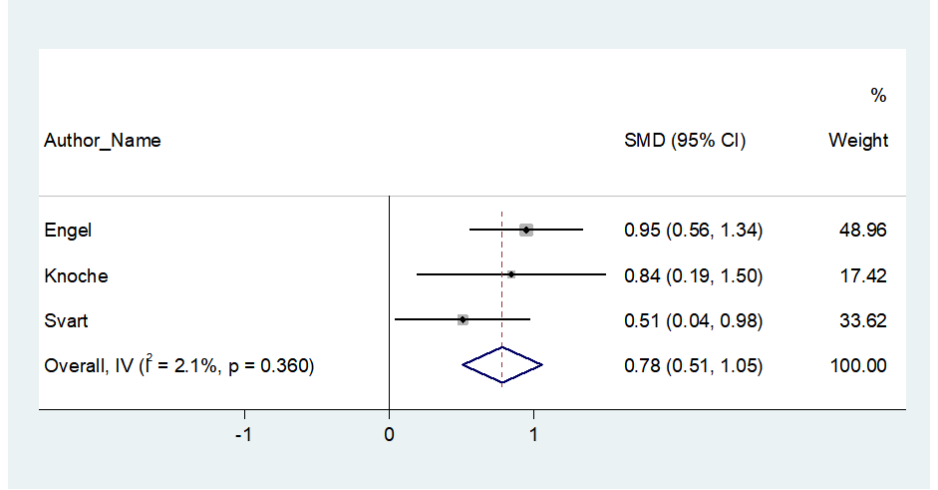
Forest plot showing the association between neurofilament – light and risk of IIH

NF-L levels were reported in three studies, including 171 IIH patients and 88 controls. Meta-analysis demonstrated a significant association between elevated NF-L levels and IIH risk (SMD 0.78; 95% CI –0.51 to 1.05). Similarly, three studies measuring plasma fibrinogen (83 IIH patients, 60 controls) indicated a significant positive association with IIH risk (SMD 0.66; 95% CI –0.08 to 1.23). These findings are summarized in the forest plots presented in Figure 2 (Fig. 2) and Figure 3 (Fig. 3).

### Secondary outcome: Association of body mass index (BMI) with risk of idiopathic intracranial hypertension (IIH)

**Figure 4:**
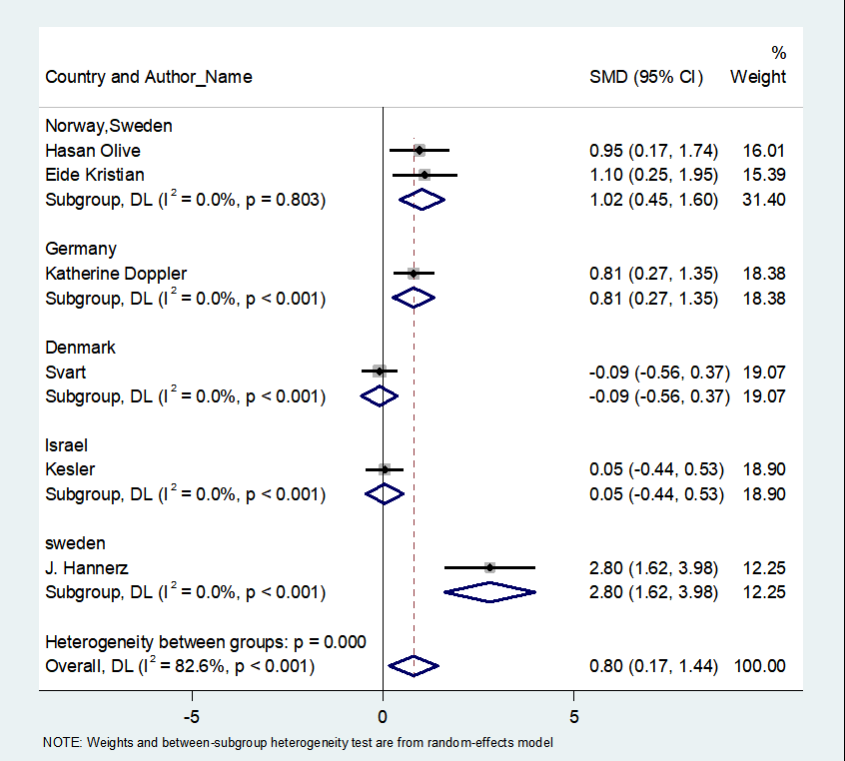
Forest plot showing the association between BMI and risk of IIH

A meta-analysis of six studies, including 137 IIH patients and 134 controls, was conducted to assess the relationship between BMI and IIH risk. The analysis demonstrated a significant association, with higher BMI correlating with increased risk of IIH in the overall population (SMD 0.80; 95% CI 0.17–1.44). The corresponding forest plot is shown in Figure 4 (Fig. 4).

### Publication bias

Funnel plot analysis and Egger’s regression test indicated no significant publication bias for the association of NF-L and fibrinogen with IIH risk. Specifically, NF-L studies (p=0.843) and fibrinogen studies (p=0.386) showed minimal bias. In contrast, analysis of BMI and IIH risk (six studies) suggested substantial publication bias (p=0.010). Results for all primary and secondary outcomes are presented in Figure S1 in Attachment 1, with Egger’s test p-values summarized in Table 2 (Tab. 2).

### Meta-regression analysis

Meta-regression was performed to evaluate whether study quality (risk of bias) influenced overall effect estimates. Results indicated that quality scores were not significantly associated with variations in effect size for any primary or secondary outcomes (Figure S2 in Attachment 1). Although heterogeneity was observed in the BMI analysis, effect sizes were independent of individual study quality. Corresponding p-values are presented in Table 2 (Tab. 2).

### Sensitivity analysis

A leave-one-out sensitivity analysis was conducted to assess the robustness of the pooled effect sizes by sequentially excluding each study. All factors affecting primary and secondary outcomes were included. As shown in Figure S3 in Attachment 1, no significant changes were observed for fibrinogen, neurofilament-L, or BMI, and no outlier studies were identified.

## Discussion

This systematic review and meta-analysis evaluated the association of gNVU) proteins – AQP4, GFAP, fibrinogen, and NfL with the risk of idiopathic intracranial hypertension (IIH). To our knowledge, this is the first comprehensive meta-analysis addressing these biomarkers in the context of IIH pathogenesis.

Our pooled analysis revealed that CSF NfL and plasma fibrinogen levels are significantly elevated in IIH patients compared to controls. NfL, a marker of axonal injury, consistently correlates with papilledema severity and CSF opening pressure, supporting its role as an indicator of optic nerve damage in IIH. Plasma fibrinogen, a key determinant of blood viscosity and a cofactor in platelet aggregation, was also elevated. Elevated fibrinogen, as an acute-phase reactant, reflects underlying inflammation and contributes to hypercoagulability, potentially increasing the risk of venous thrombosis and impairing CSF drainage.

By contrast, findings for AQP4 and GFAP were inconclusive due to methodological heterogeneity. Eide et al. reported increased perivascular AQP4 immunoreactivity and astrocytic gliosis in IIH brain biopsies, supporting the hypothesis that AQP4 dysfunction may impair water regulation. However, Doppler et al. found no difference in CSF AQP4 using ELISA, highlighting discrepancies due to sample type and detection method. Similarly, GFAP studies varied, with SiMoA assays detecting elevated CSF and serum levels, while immunohistochemical studies revealed localized gliotic changes. These differences underscore the need for standardized biomarker assessment in IIH.

Secondary analysis confirmed a robust association between elevated BMI and IIH risk, consistent with known epidemiological patterns and emphasizing the multifactorial nature of the disease. Meta-regression indicated that study quality did not significantly influence effect estimates, and sensitivity analyses confirmed the stability of these findings.

### Limitations

This review has several limitations that should be considered when interpreting the findings.

1. The selection of control groups varied widely across studies, including healthy individuals, healthy obese participants, and patients undergoing unrelated neurosurgical or diagnostic procedures, such as lumbar puncture for epilepsy or vascular disorders. Such variability introduces potential confounding factors that may influence biomarker levels independently of IIH, thereby affecting comparability between studies.
2. Most studies did not report formal sample size calculations, suggesting that several were likely underpowered to detect meaningful associations between biomarkers and IIH risk, which may have led to type II errors.
3. Methodological heterogeneity in quantifying AQP4 and GFAP further limited the ability to perform a uniform comparison. Studies differed in the type of biospecimens analyzed (CSF, serum, or brain tissue) and the techniques used (ELISA, immunohistochemistry, SiMoA, or electron microscopy), creating discrepancies in reported levels and precluding pooled quantitative analysis.
4. Furthermore, the sensitivity and specificity of the assays used to measure these biomarkers were often unreported, limiting the assessment of reliability, reproducibility, and diagnostic accuracy. The only exception was Doppler et al., who employed validated ELISA kits for AQP4 measurement, providing a more robust and interpretable dataset. Collectively, these limitations highlight the need for standardized study designs, uniform selection of control groups, consistent biospecimen types, and validated, sensitive assays in future research to strengthen the evidence base regarding gNVU biomarkers in IIH.

### Future directions

Future studies should focus on multicenter, longitudinal designs employing standardized, highly sensitive assays (e.g., SiMoA) for CSF and serum. Integrating biomarker profiling with advanced neuroimaging and clinical metrics will be critical to validate candidate biomarkers, elucidate causal mechanisms, and identify therapeutic targets within the gNVU. Such approaches may enable biomarker-guided diagnosis and personalized treatment strategies in IIH.

## Conclusion

This systematic review and meta-analysis provide evidence that gNVU dysfunction contributes to IIH pathophysiology. Elevated CSF NfL and plasma fibrinogen suggest ongoing neuroaxonal injury and a hypercoagulable state. Findings for AQP4 and GFAP remain inconclusive due to methodological heterogeneity. Collectively, these results indicate that IIH involves complex glial and vascular mechanisms beyond altered CSF dynamics.

## Abbreviations

- Aquaporin-4: AQP4
- Body mass index: BMI
- Blood–brain barrier: BBB
- Cerebrospinal fluid: CSF
- Confidence interval: CI
- Glial fibrillary acidic protein: GFAP
- Glial neurovascular unit: gNVU
- Heterogeneity statistic (I-squared): I²
- Idiopathic intracranial hypertension: IIH
- International Classification of Headache Disorders, 3^rd^ edition: ICHD-3
- Newcastle–Ottawa Scale: NOS
- Neurofilament-light chain: Nf-L
- Preferred reporting items for systematic reviews and meta-analyses: PRISMA
- Risk ratio: RR
- Standardized mean difference: SMD
- Central nervous system: CNS
- Reference: Ref

## Notes

### Authors’ contributions

- BA & MN: Study screening, data curation, quality assessment & validation
- BA & PK: Formal analysis
- BA, AKP & PT: Writing, reviewing & editing
- RKS, DV, DG, AA, DM, GP, VVY, SG, AKP: Conceptualization, formal analysis, writing – original draft

### Ethics approval and consent to participate

Ethical approval and informed consent were not required for this systematic review and meta-analysis.

### Availability of data and material

All data generated or analysed during this study are included in this published article and its supplementary information file. Data extraction sheets can be requested through email to the corresponding author. 

### Competing interests

The authors declare that they have no competing interests.

## Supplementary Material

Supplementary figures

## Figures and Tables

**Table 1:**
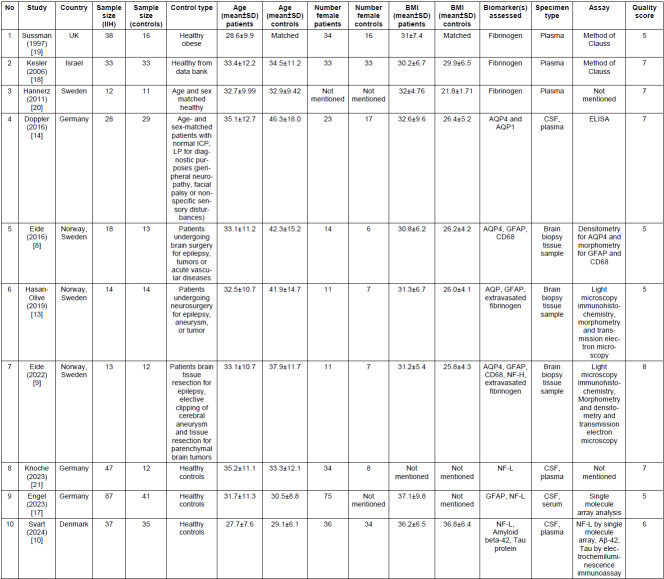
Characteristics of the included studies in the meta-analysis for the relationship between glial neurovascular proteins and risk of IIH. Assessed using the Newcastle–Ottawa Scale

**Table 2:**
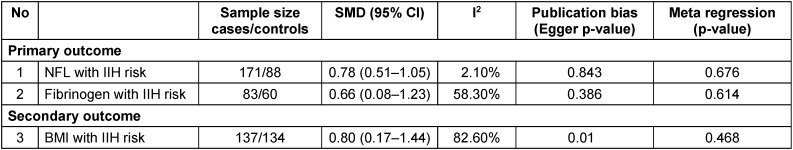
Effect size of the overall population and subgroups within the included studies of our meta-analysis
