# Supplementary material for: Glial neurovascular unit protein dysregulation and risk of idiopathic intracranial hypertension: A systematic review and meta-analysis
Source: Ger Med Sci. 2026 Jun 3;24:Doc05. doi: 10.3205/000358 (PMC13366126; doi:10.3205/000358)
Supplement: Supplementary figures [file GMS-24-05-s-001.pdf]

Attachment 1

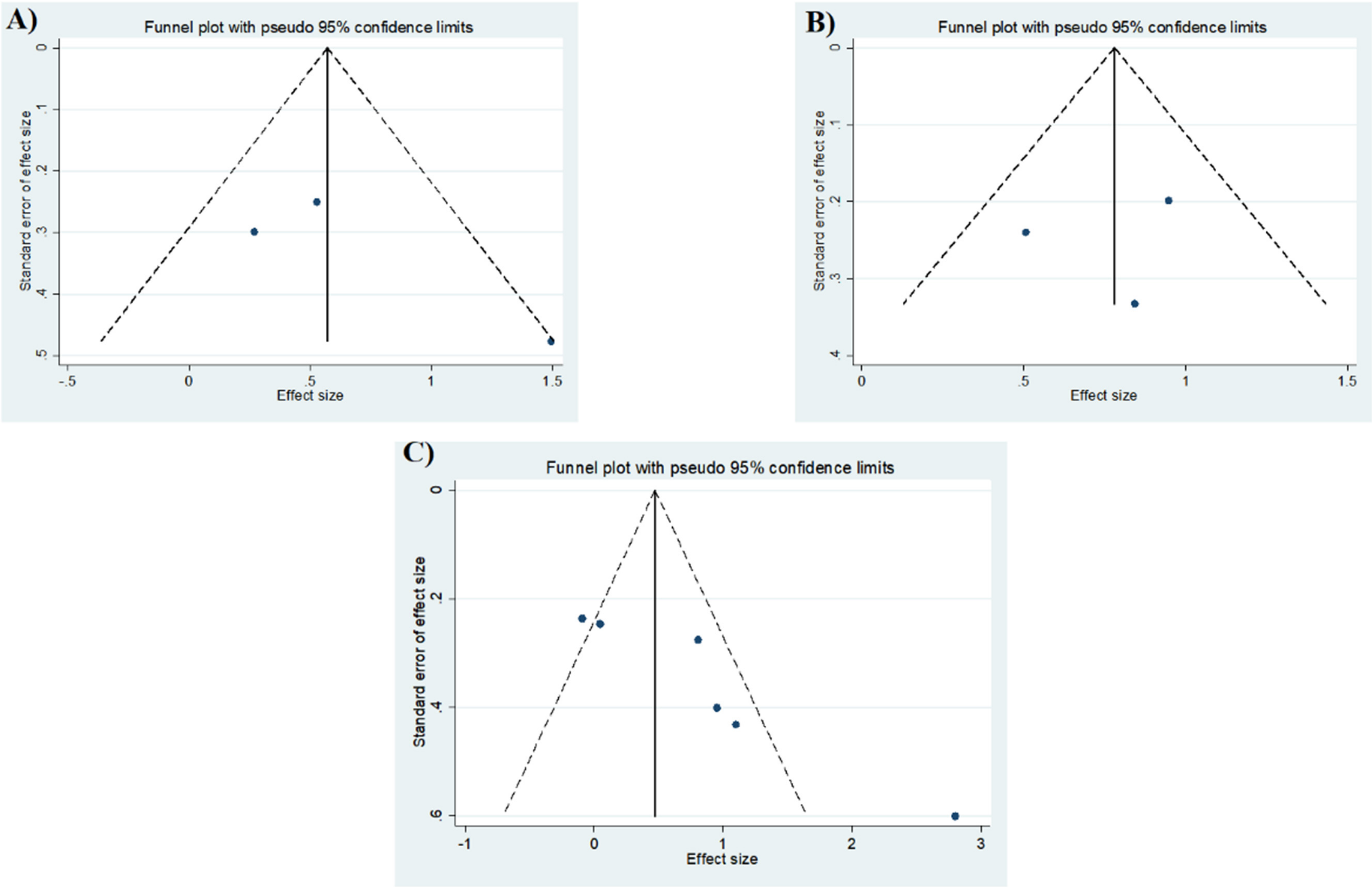

Figure S1: Begg's Funnel assessing publication bias among studies depicting the association between A) fibrinogen and risk of IIH, B) neurofilament – Light and risk of IIH, C) BMI and risk of IIH

Attachment 1 to: *Arora B, Nath M, Tangri P, Kumar P, Singh RK, Singh GP, Vibha D, Srivastava AK, Garg D, Agarwal A, M. R. D, V. Y. V, Pandit AW. Glial neurovascular unit protein dysregulation and risk of idiopathic intracranial hypertension: A systematic review and meta-analysis. GMS Ger Med Sci. 2026;24:Doc05. DOI: 10.3205/000358, URN: urn:nbn:de:0183-0003580*

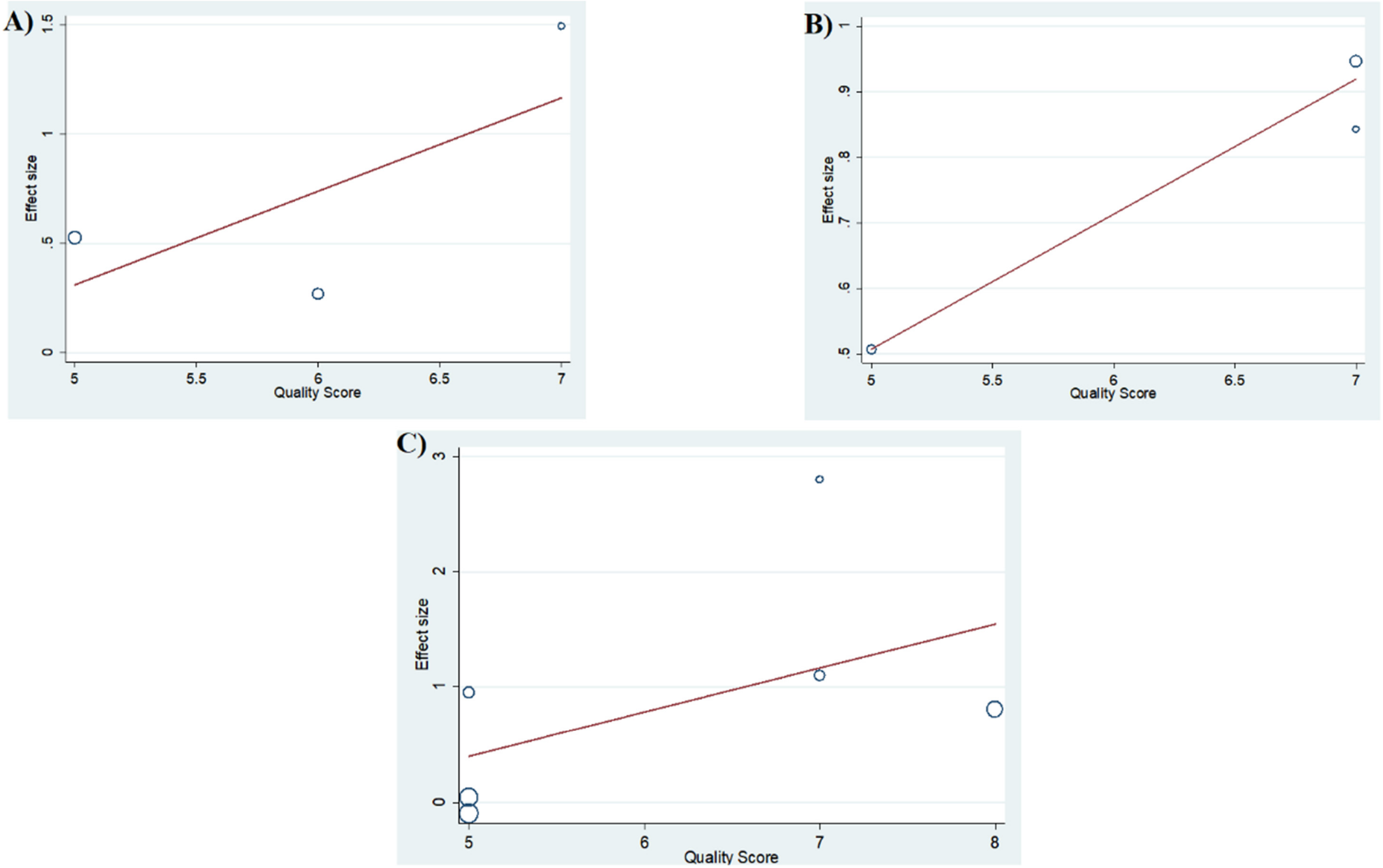

Figure S2: Meta-regression analysis of included studies to assess the effect of the association between (A) fibrinogen and risk of IIH, B) neurofilament – light and risk of IIH, C) BMI and risk of IIH

Attachment 1 to: *Arora B, Nath M, Tangri P, Kumar P, Singh RK, Singh GP, Vibha D, Srivastava AK, Garg D, Agarwal A, M. R. D, V. Y. V, Pandit AW. Glial neurovascular unit protein dysregulation and risk of idiopathic intracranial hypertension: A systematic review and meta-analysis. GMS Ger Med Sci. 2026;24:Doc05. DOI: 10.3205/000358, URN: urn:nbn:de:0183-0003580*

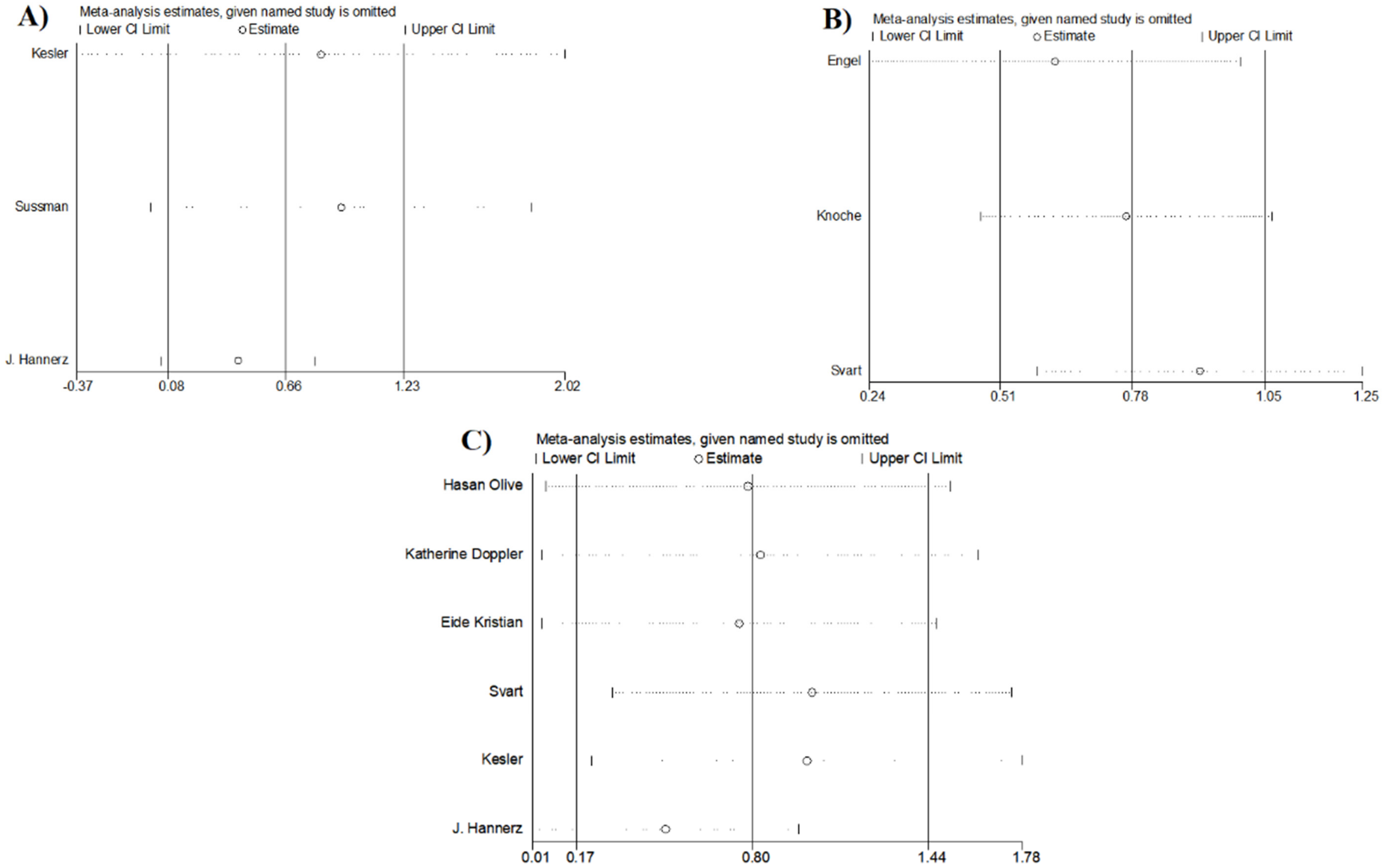

Figure S3: Sensitivity analysis plot to assess the effect of the association between A) fibrinogen and risk of IIH, B) neurofilament – light and risk of IIH, C) BMI and risk of IIH

Attachment 1 to: *Arora B, Nath M, Tangri P, Kumar P, Singh RK, Singh GP, Vibha D, Srivastava AK, Garg D, Agarwal A, M. R. D, V. Y. V, Pandit AW. Glial neurovascular unit protein dysregulation and risk of idiopathic intracranial hypertension: A systematic review and meta-analysis. GMS Ger Med Sci. 2026;24:Doc05. DOI: 10.3205/000358, URN: urn:nbn:de:0183-0003580*
